# Evaluation of Methods for the Extraction of DNA from Drinking Water Distribution System Biofilms

**DOI:** 10.1264/jsme2.ME11132

**Published:** 2011-11-10

**Authors:** Chiachi Hwang, Fangqiong Ling, Gary L. Andersen, Mark W. LeChevallier, Wen-Tso Liu

**Affiliations:** 1 Department of Civil and Environmental Engineering, University of Illinois Urbana-Champaign, 205 N. Mathews Ave., Urbana, IL 61810, USA; 2 Ecology Department, Earth Sciences Division, Lawrence Berkeley National Laboratory, 1 Cyclotron Rd., CA 97420, USA; 3 American Water, 1025 Laurel Oak Rd., Voorhees, NJ 08043, USA

**Keywords:** DNA extraction, drinking water distribution system, biofilm microbial community

## Abstract

While drinking water biofilms have been characterized in various drinking water distribution systems (DWDS), little is known about the impact of different DNA extraction methods on the subsequent analysis of microbial communities in drinking water biofilms. Since different DNA extraction methods have been shown to affect the outcome of microbial community analysis in other environments, it is necessary to select a DNA extraction method prior to the application of molecular tools to characterize the complex microbial ecology of the DWDS. This study compared the quantity and quality of DNA yields from selected DWDS bacteria with different cell wall properties using five widely used DNA extraction methods. These were further selected and evaluated for their efficiency and reproducibility of DNA extraction from DWDS samples. Terminal restriction fragment length analysis and the 454 pyrosequencing technique were used to interpret the differences in microbial community structure and composition, respectively, from extracted DNA. Such assessments serve as a concrete step towards the determination of an optimal DNA extraction method for drinking water biofilms, which can then provide a reliable comparison of the meta-analysis results obtained in different laboratories.

Biofilm growth in drinking water distribution systems (DWDS) is of public health concern. To better understand how potable water can be maintained and distributed to consumers, it is important to monitor changes in microbial communities with respect to the environmental conditions in the DWDS. For instance, several studies have investigated the effects of disinfectants (21, 29), nutrients (10), and DWDS materials (37) on microbial communities in drinking water biofilms, while others have looked at the changes in microbial communities (2, 15) and the persistence of pathogens (8, 32) throughout the distribution system. Studies on microbial communities often use molecular approaches based on phylogenetic analyses of rRNA sequences. Although microbial communities in drinking water biofilms have been characterized from various locations, no study has yet attempted to compare the efficacy of nucleic acid extraction procedures, which may affect subsequent interpretation of the microbial communities.

Many of the widely used methods have been developed for extracting DNA from soil. These methods involve procedures such as incubation during enzyme lysis and phenol/chloroform extraction, which are laborious, time-consuming, and generate hazardous wastes. Moreover, the amount of DNA obtained during phenol/chloroform extraction can differ among analysts. Unlike soil samples, which can be obtained relatively easily in large quantity, drinking water biofilms can be limited by biomass availability. The presence of humic substances and corrosion in the DWDS can also interfere with the DNA extraction process, which can inhibit the downstream PCR. Thus, methods developed for soils may not be suitable for drinking water biofilms. It is therefore necessary to determine the impact of DNA extraction biases on the analysis of microbial communities in drinking water biofilms.

The objective of this study was to determine a suitable DNA extraction method for drinking water biofilms in order to enable reproducible and reliable comparisons in subsequent meta-analysis results amongst different laboratories. We evaluated five widely used DNA extraction procedures, each with different physical, chemical, and enzymatic approaches, based on the following criteria: DNA yield, DNA purity, and the molecular weight of the extracted DNA. We then selected three out of the five DNA extraction procedures and used terminal restriction fragment length polymorphism (T-RFLP) analysis and the 454 pyrosequencing technique to demonstrate the impact of different DNA extraction procedures on microbial diversity and composition.

## Materials and Methods

### Bacterial monocultures

The different cell wall properties of bacteria may confer varied resistance to cell lysis treatments; therefore, we evaluated the quantity and quality of DNA yield from the five selected protocols using bacteria with different cell wall properties, which included the Gram-negative *Aeromonas caviae* (ATCC 14486), *Aquabacterium parvum* (ATCC BAA-207), and *Sphingomonas* sp. RO2 (bacterial isolate, University of Singapore, Singapore); the Gram-positive *Bacillus subtilis* (ATCC 23856) and *Gordonia hirsuta* (ATCC 700255); the acid-fast *Mycobacterium smegmatis* (ATCC 19420); and *Escherichia coli* (ATCC 4157) as a positive reference strain. More importantly, these bacterial strains were selected as their related species have been isolated from drinking water and are either biofilm producers or are part of the biofilm in the DWDS, and some are also opportunistic human pathogens (8, 32). Hence, evaluating the DNA yield of the protocols with these bacterial strains is important for downstream characterization of the DWDS biofilm community as well as for detection of potential pathogens from a public health perspective. The bacteria were harvested overnight and the collected cell pellets were used for DNA extraction and biomass (dry weight) determination. DNA was extracted in triplicate using each of the five different extraction methods.

### Drinking water distribution system samples

After DNA extraction from bacterial monocultures, three of the five extraction methods were selected for further analysis with DWDS samples. Biofilm collected from water meters was used to evaluate the efficiency of DNA extraction using these methods. The water meters were collected and pooled at three different times from neighborhoods in Urbana, IL. The feasibility of using biofilm collected from water meters as representative of DWDS biofilm has been demonstrated by Hong *et al.*(11). The inner components of the water meters were separated into brass and plastic, and the biofilm from these surfaces was swabbed with sterile cotton swabs. The collected biomass was suspended in 1×PBS, vortexed, centrifuged, and stored at −80°C until used. The samples were divided into aliquots such that DNA could be extracted from triplicate samples using each of the three extraction methods.

### DNA extraction and purification methods

The five DNA extraction methods included two commercial kits, the PowerSoil DNA Isolation kit (MO BIO Laboratories, Carlsbad, CA, USA) and the FastDNA Spin Kit for Soil (Q-Biogene/MP Biomedicals, Solon, OH, USA), and three standard phenol/chloroform methods, which included DNA extraction procedures for soil and sediment samples (24, 39) and marine picoplankton samples (31) (detailed in Table 1). DNA extraction using the two commercial kits was performed according to the manufacturer’s instructions. The three phenol/chloroform methods differed in the use of mechanical, chemical, and enzymatic treatments of the samples. The extracted DNA from each method was suspended in an equal volume (25.0 μL) of water. The extracted DNA was subjected to PCR. If no products were obtained, the extracted DNA was then further purified with the Wizard Genomic DNA Purification Kit (Promega, Madison, WI, USA).

### Purity, quantity, and quality of DNA

A Nanodrop 1000 spectrophotometer (Thermo Fisher Scientific, Waltham, MA, USA) was used to assess both the purity of DNA (via absorption ratios of the extracts at A_260_/A_280_) and the quantity of DNA. DNA is assumed to be free from protein contamination when the A_260_/A_280_ ratio is higher than 1.7. Since spectrophotometer measurements can be affected by contaminants (*e.g.* free nucleotides, salts, and organic compounds) and are not sensitive to low DNA concentrations, a fluorescent-based quantitation, Q-bit Quantitation Platform (Invitrogen/Life Technologies, Carlsbad, CA, USA), was also used to complement values obtained from the spectrophotometer. The quality of the extracted DNA was evaluated by observing the size of the extracted DNA fragments via agarose (0.8%) gel electrophoresis with a DNA/*Hind*III fragment ladder (Promega) as a size standard.

### T-RFLP analysis

The bacterial community structure of each sample was assessed by performing terminal restriction fragment length polymorphism (T-RFLP) using the primers 47F (5′-6 FAM-CYTAACACATGCA AGTCG-3′) and 927r (5′-ACCGCTTGTGCGGGCCC-3′). Briefly, the reactions contained 12.5 μL Bullseye Taq 2.0 Master Mix (Midwest Scientific, St. Louis, MI, USA), 1.0 μL of each primer (10 μM final concentration), approximately 1 ng μL^−1^ DNA, adjusted to a final volume of 25.0 μL with sterilized water. Thermo cycling conditions were: initial denaturation at 96°C for 3 min; 30 cycles of 30 s denaturation at 96°C, 30 s annealing at 58°C and 60 s elongation at 72°C; and a final elongation at 72°C for 7 min. DNA of some samples extracted from the laboratory protocols may require further purification via the Wizard SV Genomic DNA Purification System (Promega) in order to obtain PCR amplified products. The PCR products were treated with mung bean nuclease (New England Biolabs, Ipswich, MA, USA) at 37°C for 1 h and purified via the Wizard SV Gel and PCR Clean Up System (Promega) according to the manufacturer’s instructions. Enzyme digestion with *Msp*I (New England Biolabs) was carried out overnight at 37°C and DNA fragment analysis was performed on the ABI 3730xl Analyzer (Applied Biosystems/Life Technologies, Carlsbad, CA, USA). To determine the effects of DNA extraction methods on the resulting bacterial community composition and structure, cluster analysis was performed with the Bray-Curtis matrix using the Primer 6 (version 1.0.3) computer program (Primer-E, Ivybridge, Plymouth, United Kingdom).

### Pyrosequencing analysis

Based on the results of cluster analysis, duplicate samples that were clustered more closely together from each triplicate extraction were subjected to pyrosequencing. The extracted DNA was amplified with bacterial specific forward 515F (5′-Fusion A-Barcode-CA linker-GTGYCAGCMGCCGCGGTA-3′) and reverse 907R (5′-Fusion B-TC linker-CCCCGYCAATTCMTTRAGT-3′) primers. PCR products were gel purified according to the manufacturer’s instructions (Promega). The 454 pyrosequencing was carried out on a 454 Life Science Genome Sequencer GS FLX (Roche, Basel, Switzerland). The sequences were trimmed (resulting sequence length was an average of 375 bp), and merged alignments of the sequences aligned via the Infernal aligner from the Ribosomal Database Project (RDP) pyrosequencing pipeline (http://pyro.cme.msu.edu/) and the NAST alignment tool from Greengenes (6) were obtained via software developed by the Biotechnology Center at the University of Illinois (http://acai.igb.uiuc.edu/bio/merge-nast-infernal.html).

To determine the bacterial composition of the samples, an RDP Classifier was used for taxonomical assignments of the aligned 454 pyrosequences at 95% confidence level (http://pyro.cme.msu.edu/). The sequences (OTUs defined at genus level) were analyzed with DCA, performed via CANOCO version 4 (Microcomputer Power, Ithaca, NY, USA), to examine the similarity of the microbial community profiles. Diversity indices (Shannon Index and Chao1 estimator), at a 3% cut-off for species-level identification, of the samples were estimated via the analytical tools available from the RDP pyrosequencing pipeline (http://pyro.cme.msu.edu/).

## Results

### DNA extraction from bacterial monocultures

**Fig. 1:**
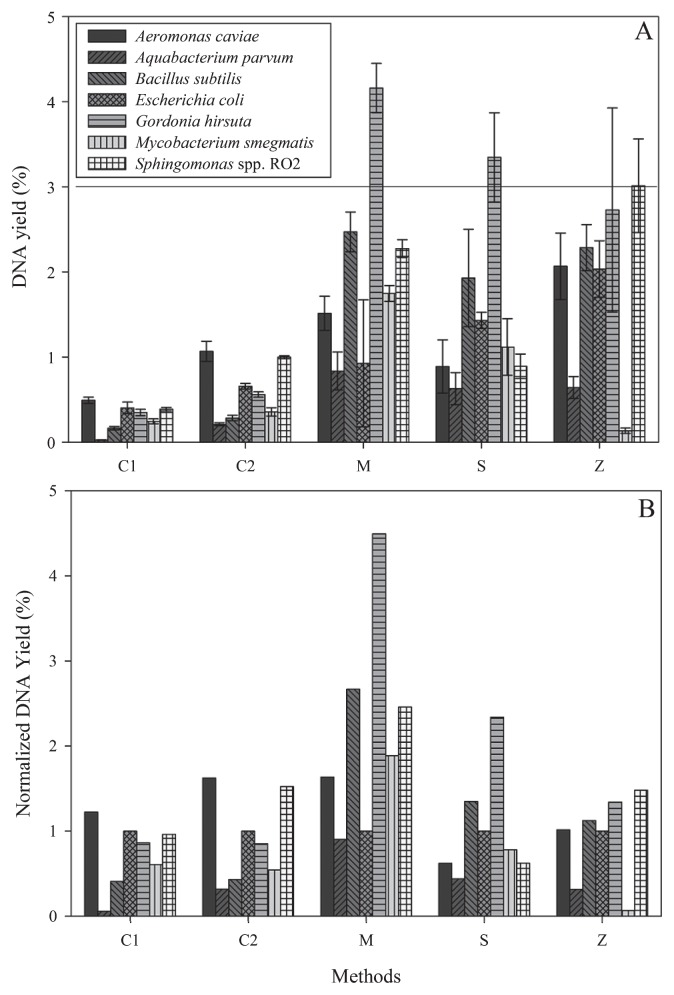
(A) DNA yield of the bacterial strains using the five methods. The solid line indicates theoretical DNA content, 3–4% of total mass (dry weight) in a bacterial cell. (B) Normalized % DNA yield to *E. coli*. Error bars indicate standard deviations of triplicate experiments.

Typically, the DNA content in a bacterial cell is ~3–4% of the total mass (dry weight). Figure 1 shows that different DNA extraction methods yielded varied amounts of DNA for the bacterial monocultures tested. Overall, the phenol-chloroform based methods yielded 4 to 5 times more DNA than the commercial kit protocols (Fig. 1A). The phenol-chloroform-based DNA extraction methods also gave higher DNA yields for Gram-positive bacteria than Gram-negative bacteria, whereas the opposite was observed when commercial kits were used. The DNA yield for the Gram-negative bacterium, *A. parvum*, was the lowest regardless of the methods used. Zhou’s protocol was the least efficient in extracting the acid-fast *Mycobacterium* (Fig. 1A). To interpret the bias introduced by DNA extraction methods, the percent DNA yield of the reference bacteria was normalized to that of *E. coli*. The commercial kit protocols and the phenol-chloroform based DNA extraction methods each showed similar trends in DNA yield (Fig. 1B). Thus the commercial kit protocols and the phenol-chloroform-based DNA extraction methods differed in extraction efficacy with respect to different bacterial species and this may be important for downstream microbial population profile analysis. The overall DNA purity from each method had an average of A_260_/A_280_ >1.8 (data not shown), which indicated that the methods were efficient in removing protein contamination.

**Fig. 2:**
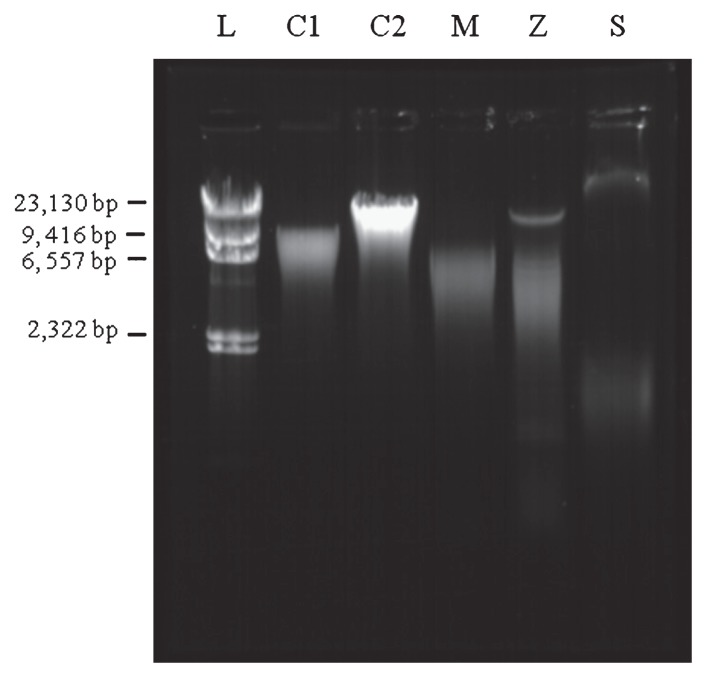
Fragment size of extracted DNA (*Sphingomonas* spp. as representative of the bacterial cultures (L: *λ/Hind* III DNA ladder). Abbreviations for methods correspond to the codes in Table 1.

The extent of DNA shearing as an indication of DNA quality was evaluated via gel electrophoresis (Fig. 2). The DNA fragments indicated that DNA extracted via PowerSoil DNA kit and Miller’s protocol had the highest degree of shearing as each method gave a fragment size of approximately 4 kb (Fig. 2). As a result, the other three protocols (FastDNA, Schmidt, and Zhou), which yielded more intact DNA, were selected and further evaluated using DWDS samples.

### DNA extraction from DWDS samples

**Fig. 3:**
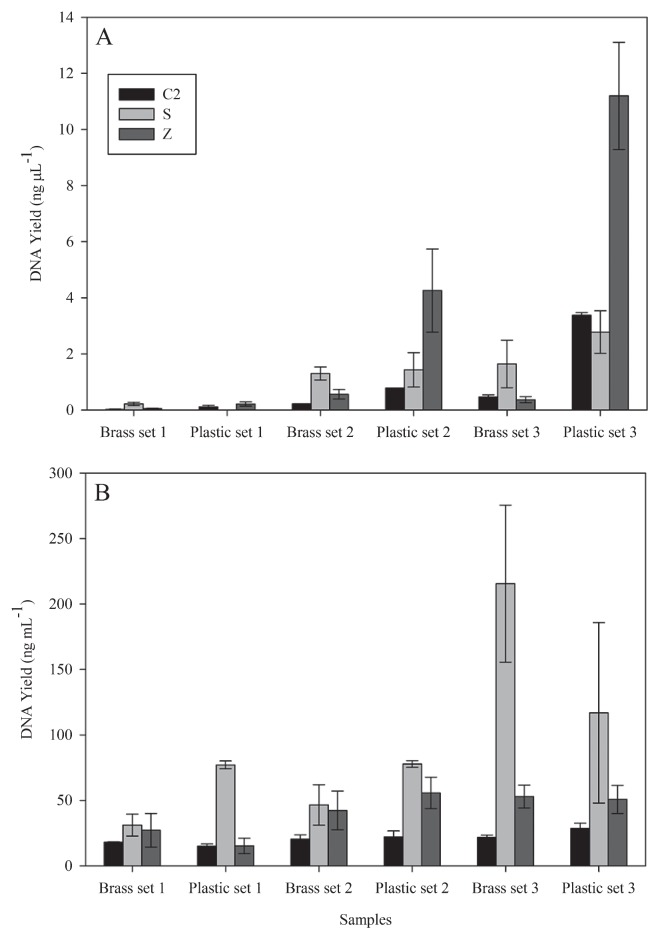
DNA yield averages of triplicate samples of water meter biofilm from brass and plastic surfaces measured by (A) Q-bit and (B) Nanodrop. Abbreviations of methods correspond to the codes in Table 1. Error bars indicate standard deviations of triplicate experiments.

The phenol-chloroform-based methods (Schmidt and Zhou’s protocol) again yielded higher DNA concentrations from DWDS samples than the FastDNA kit (Fig. 3). DNA concentration also varied between brass and plastic surfaces, which may have been influenced by surface properties or in the amount of biomass obtained from both surfaces. Since DNA extracted from each protocol showed variations in UV spectra, *e.g.* DNA extracted from FastDNA kit typically had a maximum absorbance spectrum at around 230 nm (data not shown) due to inherent kit properties, DNA concentration determined by direct spectrophotometric measurement may not be accurate. Our results showed that spectrophotometric-and fluorescent-based DNA measurements indeed gave varied DNA quantifications. DNA concentration measured by Q-bit gave a lower yield than that measured by the Nanodrop (Fig. 3), which confirmed that the Nanodrop was not sensitive to low DNA concentrations. The measured A_260_/A_280_ ratios of the DNA extracted from DWDS samples indicated that the FastDNA kit in general gave the best DNA purity. Although there were A_260_/A_280_ ratios of 1.40–1.50 for DNA extracted from some samples using the FastDNA kit, the extracted DNA could still be PCR amplified without further purification. In contrast, in some sample sets, DNA extracted using Schmidt and Zhou’s protocol required further purification in order to obtain PCR amplified products (Table 2).

### T-RFLP analysis of DWDS samples

**Fig. 4:**
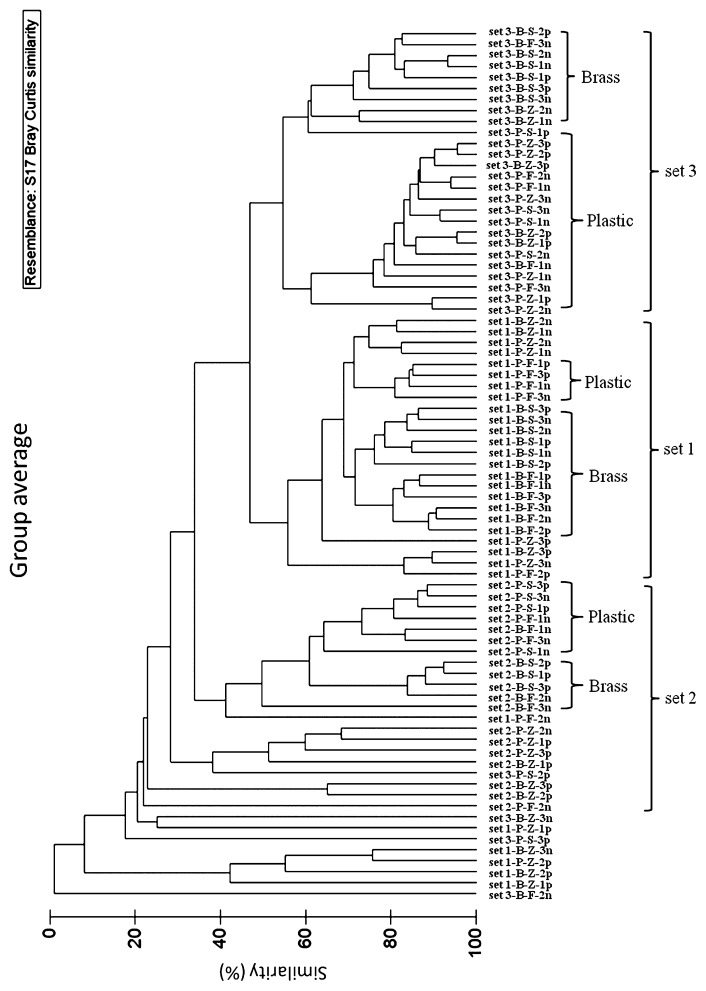
Cluster analysis constructed from similarity matrix (Bray-Curtis coefficient) representing dissimilarity of T-RFLP profiles generated from DNA samples obtained by DNA extraction methods. “F”, “S”, and “Z” refer to FastDNA’s, Schmidt’s, and Zhou’s protocols, respectively (numbers indicate triplicate extractions; “n” = before purification; “p” = after purification).

T-RFLP analysis was first used to compare the molecular fingerprinting patterns that resulted from the different DNA extraction protocols and to compare whether there were differences between samples before and after DNA purification. Cluster analysis of T-RFLP molecular fingerprinting patterns showed that the three different sample sets grouped more closely with each other. Moreover, the results also showed that samples extracted from their respective protocols were more often grouped together, which indicated that each method yielded slightly different molecular fingerprints (Fig. 4). FastDNA kit and Schmidt’s protocol also gave reproducible results as similar T-RFLP profiles were obtained from triplicate extractions of brass or plastic samples, while samples extracted from Zhou’s protocol gave varied T-RFLP profiles. In addition, FastDNA and Schmidt’s protocols indicated differences in the T-RFLP profiles between the plastic and brass samples in all three sample sets, which showed that there were different biofilm communities growing on the two surfaces. In contrast, Zhou’s protocol only indicated that there were differences in the T-RFLP profiles of the plastic and brass samples in two of the three sample sets, which suggested that Zhou’s protocol may not be as efficient as the other two protocols in discerning differences in microbial populations from different surfaces (Fig. 4).

As mentioned previously, some DNA extracted from Schmidt’s and Zhou’s protocol required DNA purification in order to obtain PCR products. Thus, DNA obtained before and after purification from some sample sets was also subjected to T-RFLP analysis in order to compare the effect of purification on T-RFLP profiles. The results showed that while purification did not affect the T-RFLP profiles in samples extracted from both FastDNA and Schmidt’s protocol, it did affect those from Zhou’s protocol as samples after purification often became less clustered with the samples before purification (Fig. 4). This showed that DNA purification may sometimes influence resulting microbial community profiles and thus the outcome of data analysis.

### Pyosequencing analysis of DWDS samples

**Fig. 5:**
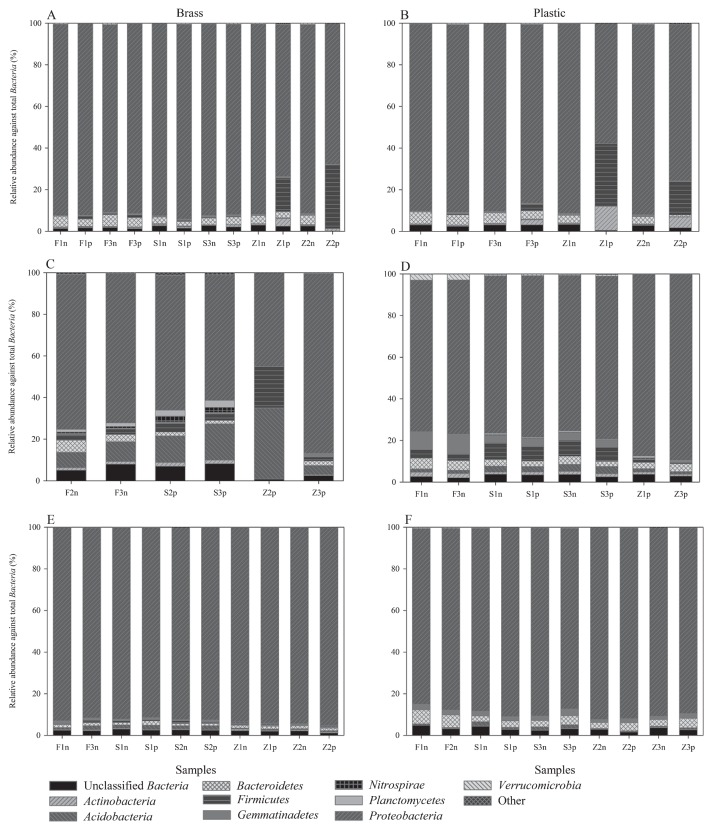
454 pyrosequencing analysis of bacterial community composition profiles at the phylum level for set 1 (A and B), set 2 (C and D), and set 3 (E and F). Bacterial phyla under the “Other” category are present at less than 1%, which include *Chlamydiae*, *Chloroflexi*, *Cyanobacteria*, *Deinococcus-Thermus*, *Fusobacteria*, *Lentisphaerae*, *Spirochaetes*, *Thermotogae*, and candidate divisions BC1, OD1, and OP10. Left and right columns indicate brass samples and plastic samples, respectively. “F”, “S”, and “Z” refer to FastDNA’s, Schmidt’s, and Zhou’s protocols, respectively (“n” = before purification; “p” = after purification).

To obtain detailed taxonomic analysis of the bacterial community composition bias associated with DNA extraction, pyrosequencing of 16S rRNA gene PCR amplicons was carried out from duplicate samples. The DWDS materials (brass and plastic) and the extraction methods affected the taxanomic composition of DNA extracts, which was apparent at the phylum and OTU levels. In the first set, the samples looked similar at the phylum level, except for the purified DNA extracts from Zhou’s protocol where an increase in the proportion of *Firmicutes* (*i.e.* predominantly *Bacillus*-like sequences) and the *Actinobacteria* (*i.e.* predominantly *Mycobacetrium*-like species) phyla was observed (Fig. 5A and B). A similar observation was also made with the second set of brass samples from purified DNA extracts of Zhou’s protocol (Fig. 5C). In contrast, the DNA extracts obtained from FastDNA and Schmidt’s protocols had sequences from different families of *Actinobacteria* and *Firmicutes* phyla (data not shown). Compared to Zhou’s protocol, the DNA extracts from FastDNA and Schmidt’s protocols gave a higher proportion of unclassified *Bacteria* and *Planctomycetes*, and *Gemmatimonadetes* in the second set of brass and plastic samples, respectively (Fig. 5C and D), and a higher proportion of *Actinobacteria* and *Firmicutes* in the third set of brass samples (Fig. 5E).

**Fig. 6:**
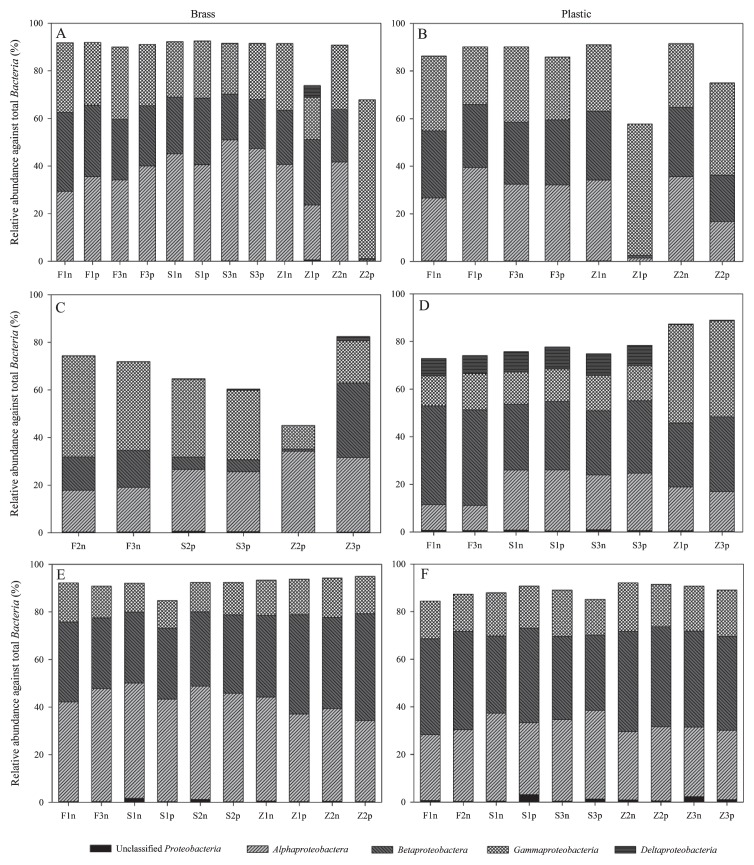
454 pyrosequencing analysis of the relative abundance of the *Proteobacteria* classes for set 1 (A and B), set 2 (C and D), and set 3 (E and F). Left and right columns indicate brass samples and plastic samples, respectively. “F”, “S”, and “Z” refer to FastDNA’s, Schmidt’s, and Zhou’s protocols, respectively (“n” = before purification; “p” = after purification).

All of the sample sets had a predominance of the phylum *Proteobacetria* (Fig. 5); however, the different extraction methods gave varied proportions of the different classes of *Proteobacteria*, *e.g. Alphaproteobacteria*, *Betaproteobacteria*, *Gammaproteobacteria*, and *Deltaproteobacteria* in the different sets of samples (Fig. 6). Closer examination of the *Proteobacterial* groups from the second set of brass samples showed that Schmidt’s protocol gave a lower proportion of *Betaproteobacteria* than FastDNA’s protocol (Fig. 6C). The second set of plastic samples also showed that both FastDNA and Schmidt’s protocols gave a higher proportion of *Deltaproteobacteria* than Zhou’s protocol (Fig. 6D). Overall, FastDNA gave more reproducible results, especially with regard to the brass samples compared to the other two protocols (Fig. 6).

**Fig. 7:**
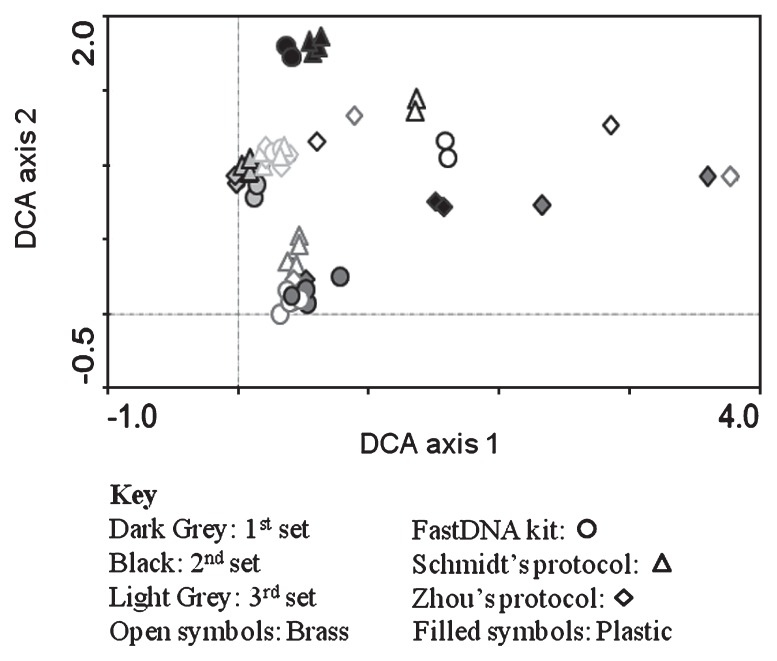
DCA analysis of bacterial communities via sequences (genus-level) obtained from 454 pyrosequencing of each sample.

De-trended correspondence analysis (DCA) of the relative abundance of the sequences at the genus level indicated significant variance associated with the sample sets (Fig. 7). The surfaces (brass and plastic) from which the biomass was collected also had different bacterial community profiles. More importantly, DCA also indicated that the different DNA extraction methods could contribute to bacterial community profile variability. In most sample sets, bacterial communities from Zhou’s DNA extracts were different than those from FastDNA and Schmidt’s (Fig. 7).

**Fig. 8:**
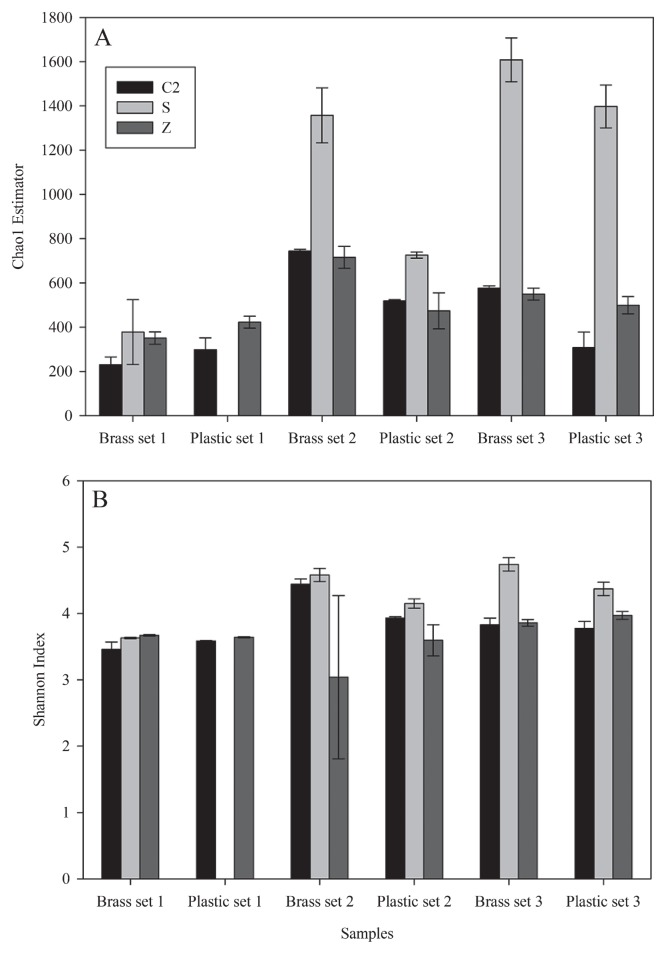
Microbial species richness (A) and diversity (B) with respect to the DNA extraction methods. Abbreviations of methods correspond to the codes in Table 1. Error bars indicate standard errors of duplicate estimations. Plastic set 1 with Schmidt’s DNA extraction method was removed from analysis due to sample contamination.

Different diversity indices have been criticized for the assumptions made on the relative importance of spread of abundance amongst species; therefore, estimations of total species richness may be a reliable alternative to provide the best approximation of actual total species richness (9). Here, both species richness and diversity were estimated in each bacterial community, using the Chao1 estimator and Shannon index, respectively. The Chao1 estimator is based on the number of species in a sample that are represented by one or two individuals, and thus is an abundance-based nonparametric species richness estimator (4). The Shannon index takes into account the number of species and the evenness of the species. Results from the Chao1 estimator and Shannon index positively correlated with each other, except for the first plastic sample sets and the third brass and plastic sample sets (Fig. 8). While results from the Shannon index seemed to indicate that diversity was similar between the samples from the different extraction protocols, Chao1 estimator showed that species richness was varied, with DNA extracts from Schmidt’s protocol usually giving the highest species richness (Fig. 8).

## Discussion

While there is a wide selection of established DNA extraction protocols, it is necessary to carefully evaluate the methods with regard to the characteristics of the sample and the intended downstream applications. Efforts to establish appropriate methods of DNA extraction from different environmental samples, *e.g.* fecal samples (1, 23), soils (7), and aquatic environments (28, 33, 34), showed that determination of an optimal method is essential to minimize biases in molecular analyses. In microbial community analysis of DWDS, the DNA extraction methods used included commercial kits developed for soils (5, 14, 37) or phenol-chloroform-based methods intended for use in different sediment types (27, 38) or planktonic microorganisms (20); however, the DWDS samples analyzed in these studies differed in whether they were actual biofilm samples, *i.e.* those obtained from DWDS surfaces, or planktonic samples, *i.e.* those obtained from bulk water. Thus, we needed to determine the appropriate DNA extraction methods for our samples, which were biofilm samples obtained from the surfaces of water meters.

The DNA extraction methods were selected based on their popularity, ease of use, cost, and differences in cell lysis methods. These methods have been tested by independent researchers for use in different applications (1, 7, 23, 25, 26, 30, 36). Our study therefore further investigated the suitability of these different DNA extraction methods for DWDS samples. The DNA extraction methods (Table 1) were first compared using biomass of bacterial monocultures. DNA fragment size, quality, and quantity were used as screening criteria to select the methods for final validation with DWDS samples. The highest molecular mass DNA was obtained using FastDNA, Schmidt, and Zhou’s protocols. Higher molecular mass DNA is desirable for PCR since larger DNA fragments reduce the chances of chimera formation during PCR (17).

Concerns with extracting DNA from Gram-positive bacteria include their relatively thicker cell wall compared to that of Gram-negative bacteria and the ability to form spores in some bacterial species. Hence, additional treatments such as chemical lysis and hot detergent have been suggested to improve spore lysis (7). Although DNA extraction with bacterial monocultures indicated that phenol-chloroform-based methods (*i.e.* Schmidt and Zhou’s protocols) had higher DNA yield for Gram-positive bacteria than Gram-negative bacteria, 454 pyrosequencing analysis results indicated that regardless of the protocols used, the proportion of Gram-negative bacteria was higher than Gram-positive bacteria in the DWDS. Moreover, 454 pyrosequencing indicated that FastDNA’s protocol yielded a similar proportion of Gram-positive bacteria in the DWDS to the phenol-chloroform-based methods even though it was not efficient in extracting DNA from Gram-positive bacterial cultures. This showed that results from bacterial monocultures may be different when applied to actual environmental samples. Here, FastDNA was able to generate comparable results to the two phenol-chloroform-based methods when applied to DWDS samples. In addition, studies have shown that the FastDNA kit was efficient in extracting DNA from samples spiked with bacterial spores (7, 26).

With bacterial monocultures, all of the DNA extraction methods yielded relatively pure DNA. When applied to DWDS samples, however, DNA purity decreased, which could be attributed to the presence of corrosion products and humic acids in the samples. The low purity of the DNA perhaps also influenced the Nanodrop measurements; therefore, DNA yield was also measured with Q-bit, whose readings indicated a much lower DNA quantity. Despite the higher DNA yield from the phenol-chloroform-based protocols, the DNA extracts from some sample sets required further purification in order to obtain PCR amplified products. In contrast, despite the low DNA yield from FastDNA, the extracted DNA was sufficient for PCR amplification because of higher DNA purity. Lear *et al.*(16) also showed that concentrations of DNA amplified by PCR were not influenced by the concentrations of the extracted DNA; therefore, it may be advantageous to use methods such as FastDNA that provided low DNA yield, but that also removed contaminants such as humic acids that inhibit PCR amplification (16).

Achieving a high DNA yield from environmental samples has been a main interest as some researchers contend that higher DNA yields would result in a more diversified pool of templates, which would then affect microbial diversity estimates; however, results from independent studies have been controversial (18). In addition, various DNA extraction protocols that gave improved DNA yield required extensive purification before DNA could be used in PCR (35, 39) and other studies have therefore set out to develop rapid DNA purification techniques (12, 35). Here, we addressed whether DNA yield and sample purification would influence sub-sequent microbial diversity and richness estimates and microbial community profiles on our DWDS samples. Our results showed that DNA purification more often led to decreased DNA concentration rather than improved yield (data not shown). Measurements of species richness and diversity varied before and after purification using Schmidt’s protocol (data not shown). This could be due to sample loss or removal of inhibitory substances, which could lead to a decrease or increase in species richness and diversity. Moreover, DNA purification altered microbial community profiles, which was especially apparent in DNA extracts from Zhou’s protocol. The microbial community profiles of DNA extracts from FastDNA and Schmidt’s protocols were less affected by purification.

## Conclusion

The results presented here suggested that the DNA extraction method of choice for DWDS samples was the commercial kit, FastDNA, despite its relative high cost in comparison to the phenol-chloroform-based methods. FastDNA’s protocol gave nucleic acids of higher purity than the phenol-chloroform-based methods and also did not generate hazardous wastes. Although phenol-chloroform-based methods gave higher DNA yields, they may require DNA purification, which became another factor in the selection of the optimal protocol for DWDS samples, especially when our results indicated that DNA purification could affect microbial community profiles. While DNA extracted from the FastDNA kit gave lower species richness estimates than Schmidt’s protocol, the microbial community composition obtained from FastDNA kit was relatively similar to that of Schmidt’s protocol and the sequences were reflective of those typically found in the DWDS. To address species diversity and richness representation, other studies suggested the use of several different validated methods in parallel and pooling the extracted nucleic acids to capture greater biodiversity (13, 22); however, others suggested that sample pooling could reduce detectable phylotype richness (19). Chandler *et al.*(3) also suggested the use of several different template dilutions during PCR if maximum diversity is desired in sample analysis. Here, we illustrated the influence of the DNA extraction method on microbial community profiles. Our results showed that, overall, the FastDNA kit was easy to use and was less time-consuming, which are desirable characteristics when analyzing a large number of samples. Most importantly, it provided representative microbial community information and reproducibility, which are important criteria to produce reliable and comparable results obtained from different laboratories. A better understanding of the microbial ecology of the DWDS is especially important from the public health perspective as the delivery of potable drinking water is crucial to human society. In order to establish efficient water treatment regimens, it is therefore essential to monitor microbial community changes in the DWDS; however, there are no standard methodologies for such analysis. Our study showed that DNA extraction is a critical step in microbial community analysis as different methods may result in different microbial community profiles, and this may also be applicable to other microbial ecology studies. We recommend that in order to better characterize microbial communities from different environments, the optimal DNA extraction method needs to be carefully selected in consideration of the sample type (*e.g.* sample availability and potential presence of contaminants inhibitory to PCR) and the overall objective (*e.g.* analyzing species diversity and richness or sample representativeness) of the experiment.

## Figures and Tables

**Table 1:** Details of the DNA extraction approach for the 5 selected methods

Method	Code	Approach
PowerSoil DNA Isolation Kit, MoBio Laboratories	C1	Mechanical (bead beating) and chemical lysis. Genomic DNA purified via solutions in the kit via spin filter columns.
FastDNA Spin Kit for Soil, Qubiogene	C2	Mechanical (bead beating) and chemical lysis. Genomic DNA purified via solutions in the kit via spin filter columns.
Miller *et al.*(1999)	M	Mechanical lysis (bead beating) and chemical lysis (high salt and high temperature incubation, 65°C for 30 min).
Schmidt *et al.*(1991)	S	Mechanical lysis (bead beating); enzymatic lysis (lysozyme and achromopetidase incubation at 37°C for 30 min); and chemical lysis (proteinase K and SDS incubation at 37°C for 2 h, followed by high salt and high temperature incubation, 60°C for 30 min)
Zhou *et al.*(1996)	Z	Chemical lysis (proteinase K incubation at 37°C for 30 min, followed by SDS, high salt, and high temperature incubation, 65°C for 2 h)

**Table 2:** DNA purity of water meter samples (from brass and plastic surfaces) evaluated by A_260_/A_280_ ratios, after DNA extraction via selected methods

Purity of extracted DNA (A_260_/A_280_)						

Methods	Brass set			Plastic set		
	
	1	2	3	1	2	3
C2	1.50±0.09	1.67±0.15	**1.71±0.03**	1.40±0.14	1.68±0.06	**1.74±0.03**
S	1.39±0.06	*1.60±0.08	1.41±0.01	NA	1.30±0.30	1.34±0.03
Z	1.57±0.04	*1.53±0.10	1.39±0.02	1.48±0.10	*1.44±0.11	1.57±0.06

Mean values and standard deviation were calculated in triplicate. Data in bold indicate ratios higher than 1.70.

* Indicate samples that require further purification in order to obtain PCR amplified products.

NA denotes samples removed from analysis due to contamination.
